# A Patient-Specific *in silico* Model of Inflammation and Healing Tested in Acute Vocal Fold Injury

**DOI:** 10.1371/journal.pone.0002789

**Published:** 2008-07-30

**Authors:** Nicole Y. K. Li, Katherine Verdolini, Gilles Clermont, Qi Mi, Elaine N. Rubinstein, Patricia A. Hebda, Yoram Vodovotz

**Affiliations:** 1 Department of Communication Science and Disorders, University of Pittsburgh, Pittsburgh, Pennsylvania, United States of America; 2 Department of Otolaryngology, University of Pittsburgh, Pittsburgh, Pennsylvania, United States of America; 3 University of Pittsburgh Voice Center, University of Pittsburgh, Pittsburgh, Pennsylvania, United States of America; 4 Center for Inflammation and Regenerative Modeling, University of Pittsburgh, Pittsburgh, Pennsylvania, United States of America; 5 Department of Critical Care Medicine, University of Pittsburgh, Pittsburgh, Pennsylvania, United States of America; 6 Department of Sports Medicine and Nutrition, University of Pittsburgh, Pittsburgh, Pennsylvania, United States of America; 7 McGowan Institute for Regenerative Medicine, University of Pittsburgh, Pittsburgh, Pennsylvania, United States of America; 8 Office of Measurement and Evaluation of Teaching, University of Pittsburgh, Pittsburgh, Pennsylvania, United States of America; 9 Otolaryngology Wound Healing Laboratory, Children's Hospital of Pittsburgh, Pittsburgh, Pennsylvania, United States of America; 10 Department of Pathology, University of Pittsburgh, Pittsburgh, Pennsylvania, United States of America; 11 Department of Surgery, University of Pittsburgh, Pittsburgh, Pennsylvania, United States of America; California Institute for Regenerative Medicine, United States of America

## Abstract

The development of personalized medicine is a primary objective of the medical community and increasingly also of funding and registration agencies. Modeling is generally perceived as a key enabling tool to target this goal. Agent-Based Models (ABMs) have previously been used to simulate inflammation at various scales up to the whole-organism level. We extended this approach to the case of a novel, patient-specific ABM that we generated for vocal fold inflammation, with the ultimate goal of identifying individually optimized treatments. ABM simulations reproduced trajectories of inflammatory mediators in laryngeal secretions of individuals subjected to experimental phonotrauma up to 4 hrs post-injury, and predicted the levels of inflammatory mediators 24 hrs post-injury. Subject-specific simulations also predicted different outcomes from behavioral treatment regimens to which subjects had not been exposed. We propose that this translational application of computational modeling could be used to design patient-specific therapies for the larynx, and will serve as a paradigm for future extension to other clinical domains.

## Introduction

The vocal folds are exposed to nearly continuous biomechanical stress during phonation. Increased intrafold contact stresses associated with certain voicing patterns can result in structural damage to the vocal fold mucosa [1], [2]. Specifically, phonotrauma can (1) alter the tissue's *physical properties* by disrupting intracellular adhesion [1], and (2) modulate the tissue's *cellular/molecular responses* by altering gene expression [3]. Persistent stress can further lead to tissue disorganization [1], [4], [5], stimulation of extracellular matrix synthesis [3], [6], and ultimately, the deposition of frank phonotraumatic lesions, dysphonia, and quality-of-life changes [7]–[10].

Our long-range goal is to generate a technology that will allow clinicians to prescribe a personalized vocal exercise (or rest) program that should optimize tissue healing in cases of both acute and chronic phonotrauma [11]–[14]. The first-line approach to the treatment of phonotrauma is usually behavioral [15]–[17]. Traditionally, behavioral voice treatment involves complete or partial voice rest with the hope that the ensuing inflammatory response will subside on its own [18]. However, our recent *in vitro* data from vocal fold fibroblast cultures and human data derived from concentrations of inflammatory mediators in laryngeal secretions suggest that contrary to clinical wisdom, some forms of vocal fold tissue mobilization—specifically mobilization in so-called “resonant voice” (roughly, classical singing) exercises may be able to modulate the inflammatory and healing process by *blunting the cells*' *pro-inflammatory responses* as well as *enhancing their anti-inflammatory responses.* From the wound healing perspective, this modulation may optimize the healing outcomes of the stressed/traumatized tissue in acute phonotrauma [19], [20]. Equally important, both *in vitro* and human clinical data suggest that the benefits of these tissue mobilization exercises for acute vocal fold inflammation are dose-dependent [19]. These observations suggest a commonality across tissue types in the response to injury, given that active rehabilitation is now used to treat many types of injuries [21]–[34]. However, details are lacking about mobilization dose that may optimize healing in laryngeal tissues, and how optimized doses may interact with the specific initial inflammatory status of the tissue. Purely empirical approaches to addressing this question are unattractive because of the relatively invasive and expensive nature of the research protocols [35]. The cumbersome nature of data collection also complicates the potential for biologically oriented clinical trials on the value of therapeutic interventions for phonotrauma in humans.

### Inflammation and Healing in Phonotrauma

Phonotrauma, like all other forms of trauma, is a highly complex process induced by a variety of stimuli, modulated by numerous cells and their products, and affecting different tissues in diverse ways. At the heart of the response to phonotrauma is the intertwined process of inflammation and wound healing [36]–[42]. Inflammation is the earliest and necessary response for the subsequent phase of wound healing [43]. The whole inflammatory process can be understood as an information system that processes and controls the biochemical signals induced by injury and/or infection. These signals include: (1) “Go” signals to initiate the inflammatory reactions, (2) “Stop” signals to temper the inflammation, and (3) “Switch” signals to convert a tissue-damaging mode to a healing mode [44]. Also, these signals are actively monitored and regulated by a number of checkpoints within the system. If any of the aforementioned signals is missing or one of the checkpoints malfunctions, the normal inflammatory process is altered possibly resulting in persistent inflammation, abnormal healing and tissue distortion. A *prompt* transition from the inflammatory phase to the healing phase is a key determinant of good wound healing, that is the replacement of traumatized tissue by healthy tissue [45].

Wound healing outcomes depend on the original insult to a certain degree, and are also likely influenced by individual genetics. Under ideal conditions, the inflammatory response would be limited by the antagonistic interactions among the various pro- and anti-inflammatory agents, followed by a transition to the later healing process in an orderly fashion. However, if repeated injuries occur over a period of time, the normal healing process would be disrupted. Two possible healing processes can occur in that case, namely, *reparative regeneration* and *constructive repair*. Reparative regeneration is *scarless* wound healing, in which traumatized tissue is completely restored in normal architecture and function. On the other hand, constructive repair is more common in humans. The lost tissue is replaced by granulation tissue which matures into a scar. Depending on the amount of scarring, scarred tissue usually does not properly restore the architecture and perform the pre-morbid function of the injured tissue. The vocal folds are generally capable of withstanding phonatory stresses and have the reparative capability of resolving microscopically phonotraumatic damage incurred during daily voice use. However, when injury exceeds a critical threshold, inflammation is usually clinically evident and constructive repair is involved. As a result, macroscopic vocal fold lesions may develop, with prolonged dysfunction in vocal fold vibratory function and voice output characteristics [46], [47].

Our research team has published four studies on the development of a novel method for obtaining quantitative information about the inflammatory status of the larynx from laryngeal secretions. Our prior studies [48], [49] demonstrate the presence of various mediators (interleukin-1 beta [IL-1β], tumor necrosis factor alpha [TNF-α]), prostaglandin E2 [PGE-2], and matrix metalloproteinase 8 [MMP-8] ) in vocal fold surface secretions of human subjects in response to phonotrauma. Our first study showed that pre- to post-vocal loading shifts in mediator concentrations were clearly evident at 10 and 20 min post-loading for IL-β, TNF-α, and MMP-8, reflecting the presence of acute phonotrauma. In contrast, concentration shifts were not shown for TGF-β1 or PGE-2 [49]. Another, intraoperative human study confirmed that IL-1β was an indicator of acute inflammation, whereas PGE-2 characterized chronic wounds [48]. A third study showed that IL-1β was an early indicator of inflammation, and PGE-2 was a later indicator of wound healing in rabbits subjected to surgical trauma, with IL-1β returning to baseline by Day 7 post-injury and PGE2 remaining elevated until the final time-point of three weeks post-injury [50]. Finally, a fourth study in rabbits subjected to surgical trauma assessed the degree to which assays of laryngeal secretions may reflect wound healing processes deep to the epithelium [51]. That study showed that the time point associated with spikes in IL-1β (24 hours) corresponded to the presence of fibrinous clot. The time point associated with maximum PGE-2 levels (7 days) was associated with the presence of mature collagen. Massive cellular infiltration and complete epithelial coverage were found at intermediate time points. Taken together, these studies provide robust evidence that secretions from the laryngeal surfaces can provide a quantitative reflection of the current inflammatory and wound healing state of vocal fold tissue. The attractiveness of this marginally invasive technology is that it can be readily used in human subjects—although not without some difficulties on the part of both subjects and examiners–and thus the data gain external validity over data obtained from more invasive technologies involving animal subjects. Our research team has also examined the potential of mathematical modeling to elucidate apparently contradictory and unpredictable behavior emerging from the plethora of interactions among biologic pathways involved in the acute inflammatory response [52]–[54]. We have recently coined the term “translational systems biology” to refer to the process of creating, calibrating, and validating computational simulations in settings of complex diseases, simulations that are designed *a priori* for the purpose of modifying clinical treatment, carrying out *in silico* clinical trials, generating novel therapies, and refining diagnoses [55], [56].

The objective of the present study was to use empirical data from human subjects to develop a mechanistic computational simulation for the biological dynamics of vocal fold inflammation and wound healing following acute phonotrauma. The modeling approach employed in the present study involves Agent-Based Models (ABMs). In such models, individual components of a given complex system interact based on rules whose outcomes are partially determined by stochastic processes [57]. More specifically, ABM involves discrete event simulation to study the behavior of complex systems. ABM is the most direct initial approach to simulate the temporal evolution of a complex system and to encode complicated time-dependent cellular and molecular events that occur during inflammation and wound healing [58]–[61]. Our results suggest that patient-specific, mechanistic, and predictive computational simulations of inflammation can be generated, raising the possibility that similar methods could be utilized in other complex diseases.

## Results

### Overview

**Figure 1:**
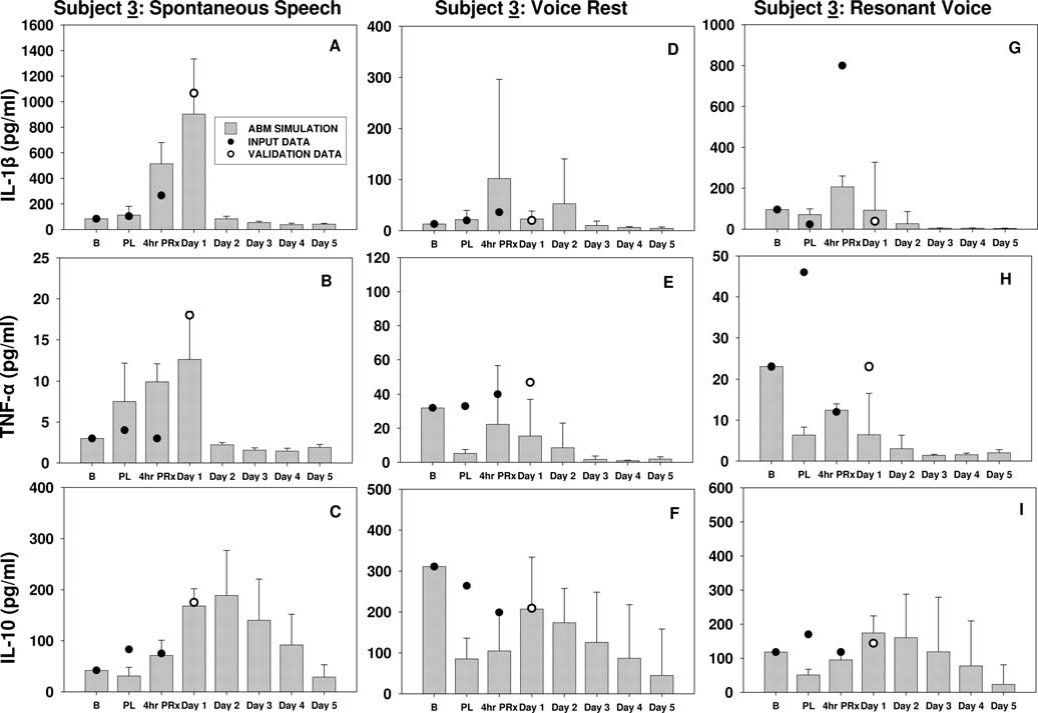
Empirical and model-predicted inflammatory and wound healing responses to acute phonotrauma in a single human subject (Subject 3) following spontaneous speech (Panels A–C), voice rest (Panels D–F) and resonant voice treatment conditions (Panels G–I). Panels A, D and G display empirical and predicted trajectories of IL-1β. Panels B, E and H show empirical and predicted trajectories of TNF-α. Panels C, F and I show empirical and predicted trajectories of IL-10. Inflammatory marker concentrations are in pg/ml. The grey bars represent the mean of the simulated data, and the error bars represent standard deviations in the simulated data. The dark circles represent the input data for the first three time-points (baseline, post-loading, 4-hr post treatment onset), obtained from human laryngeal secretion data. The empty circles represent the validation data at the 24-hr time point from the human laryngeal secretion data. B: baseline; PL: post vocal loading; 4hrPRx: following a 4-hr treatment. Note that human validation data for Days 2–5 have not yet been generated.

**Figure 2:**
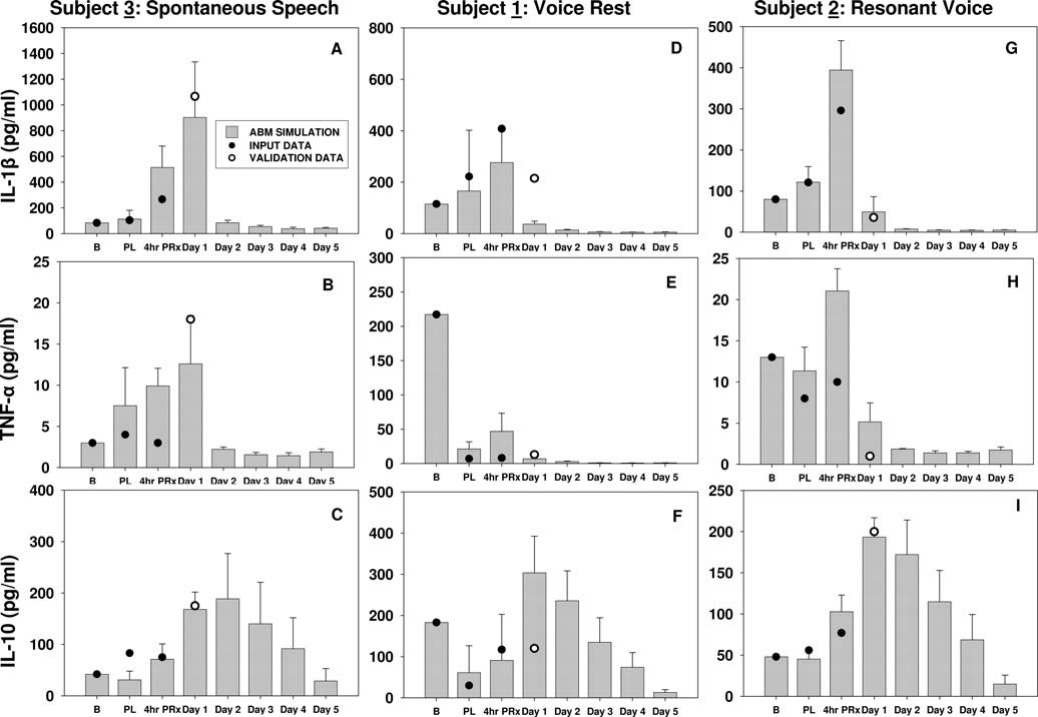
Empirical and model-predicted inflammatory and wound healing responses to acute phonotrauma in three subjects following spontaneous speech (Subject 3; Panels A–C), voice rest (Subject 1; Panels D–F) and resonant voice treatment conditions (Subject 2; Panels G–I). Panels A, D and G display empirical and predicted trajectories of IL-1β. Panels B, E and H show empirical and predicted trajectories of TNF-α. Panels C, F and I show empirical and predicted trajectories of IL-10. Inflammatory marker concentrations are in pg/ml. The grey bars represent the means from the simulated data, and the error bars represent the standard deviation from the simulated data. The dark circles represent the input data for the first three time-points (baseline, post-loading, 4-hr post treatment onset) from the human laryngeal secretion data. The empty circles represent the validation data at the 24-hr time point from the human laryngeal secretion data. B: baseline; PL: post vocal loading; 4hrPRx: following a 4-hr treatment. Note that human validation data for Days 2–5 have not yet been generated.

The premise of mathematical models involves experimental validation and feedback between the models and experiments. The details of our model calibration and validation are described in Materials and Methods. In brief, we first calibrated the ABM using data from a base cohort (Subjects 1, 2 and 3), using their mediator levels in laryngeal fluid at baseline, immediately after phonotrauma induction, and following a 4-hr treatment that involved either voice rest, “resonant voice” exercises, or spontaneous speech) (Figures 1–2, dark circles) [35]. The calibrated ABM was run 10 times for the full cohort of 7 subjects, up to 5 simulated days post baseline under the condition of (1) each subject's actual treatment group and (2) hypothetical randomization to either of the other two treatment groups, i.e., hypothetical treatments, using each subject's baseline mediator profile. Thus, a large simulation data set of subject-specific mediator trajectories (3 treatment conditions×10 runs×7 subjects = 210 runs) was generated for evaluating benefits of behavioral treatments for acute phonotrauma.

Quantitative validation of the ABM was carried out by comparing the predicted mediator levels with the empirical mediator levels at 24 hr (1) for the base cohort (Subjects 1–3), as well as (2) for Subjects 4–7 whose data had never been used for the model calibration, under the actual treatment condition. The validation results showed that predicted mediator levels generally matched empirical mediator levels for the ABM at 24 hr post baseline. Also, results from the simulation data showed that the predicted mediator trajectories varied with a function of treatment and initial mediator profile.

### Predicted Trajectories of Inflammatory Mediators

The ABM reproduced and predicted subject-specific mediator trajectories (Figures 1 and 2). In the base cohort (Subjects 1–3), the ABM predicted 24-hr mediator values in 80% (12/15; *p*<0.05) of instances (+9 mediator measurements from 3 between-group subjects, each with one of the 3 different treatments; +9 mediator measurements from one within-group subject—Subject #3– who received each of the treatments; -3 mediator measurements to account for double-counting the “spontaneous speech” treatment for Subject #3 in both between- and within-group measurements; hence 15 different mediator measurements in total). For Subjects 4–7, *z*-tests (model data vs. empirical data) indicated that the ABM predicted empirically obtained 24-hr mediator values 67% (2/3; *p*<0.05) of the times for markers that were considered “valid,” as described shortly, and 44% (4/9; *p*<0.05) of the time for markers that were considered “pre-inflamed and/or non-responsive” (see *Materials and Methods*).

For the single within-group subject (Subject 3; Figure 1), both empirical data and simulation results showed that the concentrations of pro-inflammatory mediators (IL-1β and TNF-α) spiked immediately following 1 hr of vocal loading, whereas the anti-inflammatory mediator (interleukin-10 [IL-10]) showed a more protracted course. The model predicted that following the 4-hr treatment, inflammatory mediators would have distinctive temporal and quantitative expression patterns across treatment assignments. For the spontaneous speech condition, the ABM predicted that the inflammatory response would be further escalated, i.e., would involve markedly increased secretion of both pro- and anti-inflammatory mediators following the 4-hr treatment. The concentrations of pro-inflammatory mediators (IL-1β and TNF-α) reached their peaks at Day 1 post-injury and resolved to baseline concentrations around Day 2–3 post-injury. The model also predicted that the anti-inflammatory mediator, IL-10, would be secreted in great quantities by wound macrophages during the first 5 days post-injury. On the other hand, under conditions of voice rest and “resonant voice” exercises, the predicted concentrations of the pro-inflammatory mediators dropped rapidly after the 4-hour treatment and then remained low at the end of simulation, i.e., Day 5. The anti-inflammatory mediator IL-10 was predicted to be secreted rapidly after the 4-hr treatment and remain elevated up to Day 3 post-injury. Similar mediator patterns were found in the larger dataset from the between-group subjects (Figure 2).

### Predicted Outcomes of *In Silico* Therapies

In order to demonstrate the application of ABM for running *in silico* (“virtual”) clinical studies, we ran a series of simulations for the full cohort of subjects under the conditions of (1) actual treatment received and (2) hypothetical treatments. The simulation results showed theoretical individual-specific trajectories of mediator levels across the treatments. For between-group comparisons, linear mixed models showed significant treatment effects on the predicted levels of mediators (IL-1β: F (2, 1260) = 223.17, *p*<0.001; TNF-α: F (2, 1260) = 12.27, *p*<0.001; IL-10: F (2, 1260) = 215.65, *p*<0.001). Post-hoc pairwise comparisons with Bonferroni adjustments showed that among the three treatment groups, the predicted level of the pro-inflammatory marker IL-1β ensuing from *spontaneous speech* was the highest (*p*<0.001), whereas concentrations of the same marker in the *resonant voice* condition was the lowest (*p*<0.001). At the same time, among the three treatment groups, the predicted level of the anti-inflammatory marker IL-10 ensuing from *voice rest* was the lowest (*p*<0.001), whereas the level for the resonant voice condition was the highest (*p*<0.001).

## Discussion

We asked several experimental questions in the current study. First, we sought to determine if an ABM could offer an individualized prediction of biological readouts presumed to be closely related to tissue status. Second, we wished to know if the simulated wound healing response would vary specifically as a function of the initial settings of mediator level and treatment prescribed. Positive answers to these experimental questions would strengthen confidence in the eventual utility of mathematical modeling in general, and of ABM in particular, for understanding the complex vocal fold wound healing system and for predicting the healing outcome after phonotrauma and varied treatment approaches.

The current study describes the development of an ABM that reproduced diverse trajectories of inflammatory mediators in the laryngeal secretions of different human subjects at early time-points up to 4 hrs post-phonotrauma, and furthermore was capable of predicting the levels of these mediators at 24 hrs. The subject-specific ABMs were further used to explore the effects of treatment regimens to which subjects had not been exposed and the predicted levels of mediators under each condition were compared. The models predicted that the wound healing outcomes as informed by the mediator trajectories would be dramatically different given variations in the initial mediator profile, presumably due to some combination of pre-existing vocal fold conditions and the treatment prescribed following phonotrauma.

Contemporary therapeutic interventions in phonotrauma are oriented towards modulating the inflammatory and healing processes to promote reparative healing of the traumatized vocal folds. A plausible approach is to both blunt the inflammatory response and activate the healing program. Upon mechanical challenge, an acute inflammatory cascade is immediately activated in damaged or stressed tissues [62], [63]. The inflammatory cells infiltrate the area of injury to remove damaged and dead cells and tissue debris. This inflammatory reaction contributes to additional tissue damage and cell death, which exacerbate the initial tissue damage and amplify the signals for scarring. Theoretically, inflammation-blocking interventions may reduce the “secondary” tissue damage and the possibility for fibrosis and scarring. At the same time, this approach may potentially reduce the supply of growth factors and cytokines from the inflammatory cells that facilitate tissue repair. Thus, a therapeutic balance between the need to limit inflammation causing tissue damage and the need for inflammation to initiate tissue repair is important to optimize the quality of healing outcomes and the recovery of physiological functions [62]–[66]. In addition to *in vitro* and human data from our laboratory, the current *in silico* study also suggests that modified tissue mobilization exercise in the form of “resonant voice production” may have the effect of blunting the cells' pro-inflammatory responses (e.g. IL-1β and TNF-α) but enhancing their anti-inflammatory responses (e.g. IL-10), which may ultimately promote tissue regeneration [6], [20]. At this point, only the ABMs' molecular outputs were calibrated with experimental data, whereas the ABMs' tissue-level outputs are yet to be experimentally validated. We have embarked on the work of evaluating the “net effect” of the complex interactions among these inflammatory mediators at the *tissue* level. The success of this work will lead us to predict actual vocal fold *tissue status* following injury, which ultimately may advance understanding of inflammation and healing in a clinically useful way.

Our ABM predicted that the secretion of pro-inflammatory mediators would be both prolonged and elevated subsequent to spontaneous speech following an episode of phonotraumatic injury. This prolongation would be due to a positive feedback loop involving “Pro-inflammation → Damage → Pro-inflammation,” [67] thereby delaying the transition to the subsequent healing process. Clinically, visible vocal fold inflammation would be expected. On the other hand, under conditions of either voice rest or resonant voice, the ABM predicted that the pro-inflammatory response would be attenuated. It also predicted that the anti-inflammatory response may be escalated in response to the tissue mobilization involved in resonant voice—and the pro-inflammatory response attenuated by the limitation of impact stress—thereby further prompting rapid healing [6]. These predictions suggest that the repair process would bypass the “Pro-inflammation → Damage → Pro-inflammation” positive feedback loop and avoid a full-scale inflammatory and repair response. As a result, a reparative regeneration (as opposed to constructive repair) of mucosal structure and function would be observed clinically and the probability of scar formation would be minimized in the long run.

As additional data are acquired, the current ABM is under continuous revision and augmentation. Our ultimate, long-term goal is to generate *in silico* models that can be queried to identify biomechanical treatments that will optimize the wound healing process in the vocal folds, as a function of patient-specific inflammatory profiles. Although these results are encouraging in terms of the potential translational utility of ABM in the setting of vocal fold inflammation, at least five limitations can be noted in our study.

First, the current ABM mainly simulated (1) inflammation, (2) proliferation and (3) collagen formation. The model did not account for a final phase of the wound healing process, which involves extracellular matrix (ECM) reorganization. According to the literature on dermal wound healing, ECM reorganization is initiated once a neo-matrix involving materials such as collagen is deposited at the wound site [68]–[70]. Collagen is indeed a core component of the ECM, and undergoes remodeling that is dependent on both continued collagen synthesis and compensatory collagen degradation. The degradation of wound collagen is controlled by collagenases and other proteolytic enzymes, and the net increase in wound collagen is determined by the balance of these opposing mechanisms. Compared to the large body of literature on dermal wound healing, research on ECM reorganization in vocal fold wound healing is sparse. No *in vivo* measurement of collagen remodeling in human vocal folds is currently available. Thus, in its use of empirical data obtained from non-destructive methodologies only, the current model did not incorporate aspects of collagen remodeling that might prove to be important.

A second limitation in the current study is that healing outcomes in this ABM were primarily informed by interactions among inflammatory mediators and cells. However, a growing literature supports the idea that several ECM components such as fibronectin, hyaluronic acid, and decorin could also be involved in regulating the wound healing process. Studies to date have shown that aberrant scarring/fibrosis is at least partly due to the response of fibroblasts in the wound to both inflammatory mediators and extracellular matrix components [3], [71]–[74], some of which are known to constitute alarm/danger signals (e.g. fragments of hyaluronic acid); [75] and therefore may be currently abstracted under the “damage” variable in our model. Future iterations of the ABM could be augmented with rules developed around the interactions among inflammatory mediators, cells and ECM components to yield more precise predictions.

Third, our model assumes that biomechanical stresses during phonation cause mucosal damage. However, the current biologically-based ABM lacks the ability to receive input from physical models of phonation, because data are lacking regarding the link between the output of physical models—i.e. distributed tissue stresses [76]–[79] and the biological consequences of those physical stresses. Although biochemical networks may be reasonably modeled by using stochastic simulations, many cell biology phenomena relating to wound healing require the calculation of biophysical processes, such as tissue deformation and disruption of intracellular adhesion [1]. Ideally, our synthesized biochemical networks should be coupled with these biophysical processes to yield a more complete picture of vocal fold wound healing in response to biomechanical stresses during phonation.

A fourth limitation relates to the inclusion of the multi-functional anti-inflammatory mediator TGF-β1 in our model, although our earlier work on assaying biochemical markers of vocal fold wound healing failed to detect this mediator in laryngeal secretions pre- or post-traumatically [48]. TGF-β1 is known to be involved in the regulation of cell proliferation, cell differentiation and extracellular matrix formation in all phases of inflammation and wound healing [69], [80]. This mediator exerts both anti-inflammatory and pro-fibrotic effects that could convert an active site of inflammation into a site dominated by subsequent tissue repair [81]. We suspect that TGF-β1 might be highly cell-associated [82]–[84], and that this property might have led to our inability to detect this growth factor in vocal fold secretions [48]. To determine if TGF-β1 is necessary for a correct simulation of inflammation and wound healing in the vocal folds, a qualitative validation procedure was carried out to determine what the simulated data would show in the presence or absence of TGF-β1. In the latter case, the ABM predicted that no cellular or molecular events would be triggered for any ranges of initial damage (data not shown). In the presence of TGF-β1, the ABM predicted different inflammatory and wound healing curves that vary with initial mediator profile and treatment modalities following phonotrauma (Figures 1 and 2). These results indicated that TGF-β1 (or a qualitatively similar cytokine) is essential for the wound healing process and should be included in the ABM structure.

The fifth, and perhaps most important limitation concerns the validation of the ABM predictions with regard to the different treatment modalities. We have in this study shown data from seven humans and the capacity of our ABM to predict mid-term (24-hr) inflammation based on earlier short-term assays. The subject population in this study was relatively homogeneous in terms of age. The subjects were all relatively young (21–46 years) and it is known that tissue regenerative processes usually decline with aging. We have carried out preliminary mathematical modeling of the effects of aging on the acute inflammatory response [85]. We have not, however, verified in a large cohort of patients the validity of ABM predictions with regard to treatment outcome. It should be noted that such studies in humans are complex from regulatory, practical, and ethical points of view. We have embarked on a larger clinical study to validate the ABM described herein. Indeed, clinical management of phonotrauma remains a challenge to clinicians. Large clinical trials are needed to establish optimized patient-specific treatment interventions. In addition to validating the ABM with further experimental studies, a parallel development of ordinary differential equation-based models (EBMs) has been pursued in the interest of cross-platform comparison of results [86]. This “model docking” is a well-vetted validation strategy based on a comparison of predictions of different models across an array of user input data. The finding of similar predictions in ABMs and EBMs would increase confidence in the underlying assumptions in the current ABMs. Also, the EBMs alone allow formal mathematical analysis of the simulation results, such as, bifurcation and stability analysis, that will facilitate capture of the systems dynamics of the inflammatory and healing responses [60].

Finally, for the approach ultimately to be useful in a real-life clinical setting, the time lag introduced by ELISAs will have to be addressed, or an alternate methodology will have to be identified that can yield biological profiles of materials in a shorter period of time. In the meantime, there is encouragement that models such as ABMs and EBMs have potential to illuminate important information about individualized wound healing processes in the larynx.

More globally, we suggest that a systems biology approach that involves modeling is integral to sorting through the perplexing array of factors that dictate success or failure of clinical trials for complex diseases [87]. Ultimately, this process would be augmented by the inclusion of genetic variability in inflammatory and wound healing components, typically mediated via single-nucleotide gene polymorphisms in relevant genes [88].

In summary, this study suggests for the first time that patient-specific, individualized models of inflammation and healing are possible. This demonstration extends the power of translational simulations of acute inflammation beyond the responses of idealized organisms [89]–[91], quantitative prediction of inflammation occurring in experimental animals [52], and simulations of populations (clinical trials) [88], [92], [93]. It is hoped that this work will point the way to addressing other complex disease processes.

## Materials and Methods

### Experimental Protocol for Acute Phonotrauma in Different Treatment Modalities

The study was approved by the Institutional Review Board at the University of Pittsburgh. A total of nine subjects participated in the study; six females (21–46 years) and three males (21–29 years). Eight of nine subjects participated in a between-subjects study design, which involved exposure to one “treatment” condition (voice rest, “resonant voice” exercises or spontaneous speech) following a vocal loading task. One female subject (Subject 3) was involved in a within-subjects design, and received all three treatments, randomly ordered without replacement on different pairs of days separated by intervals ranging from 1–6 months. Prior to subjects' participation in the experimental part of the protocol, written informed consent was obtained by an investigator or research coordinator and then subjects received a screening in the clinic for gag response and nasal patency. Exclusion criteria for the study included gag reflex with tooth-brushing or history of exaggerated gag reflex, deviated septum (based on the otolaryngology exam), current or recent voice problems (within 1 year), current or any history of speech or language deficits, current use of drugs that may influence the voice (e.g., diuretics, decongestants), and allergy to local anesthetics (especially lidocaine).

In this experimental protocol, a vocal loading task aimed to induce an acute phonotrauma or acute laryngeal inflammation. The protocol for vocal loading entailed three consecutive cycles, each involving 15 minutes of loud phonation (∼75–90 dB @ 15 cm microphone-to-mouth distance) followed by 5 minutes of silence, for a total 60 minutes. Then, the subjects were randomly assigned to one of three treatment groups: voice rest, “resonant voice” exercises or spontaneous speech for 4 hours in the clinic, under the careful supervision of a voice trainer, who-for the majority of subjects-was blinded to the experimental hypotheses. These three treatment modalities can be considered on a continuum of tissue mobilization magnitude and as important, intra-vocal fold impact stress magnitude: no mobilization or impact stress (voice rest), normal-to-large magnitude but relatively low impact stress (“resonant voice” exercise; [94], [95]) and normal to larger magnitude mobilization but potentially greater impact stress, depending on the mode of phonation (spontaneous speech; [94], [95]). The “resonant voice” exercise condition involved cycles of 4 minutes of voice exercise using “resonant voice,” defined as “easy” voice associated with perceptible anterior oral vibrations [96]followed by 16 minutes of rest, whereas the spontaneous speech condition involved repeating cycles of 16 minutes of conversational speech followed by 4 minutes of silence. After a 4-hour treatment period monitored in the clinic, participants were discharged to home with instructions to continue their corresponding treatments (in somewhat less intense cycles for resonant voice and spontaneous speech conditions). The following morning, participants were required to observe complete voice rest until their arrival at the clinic between 7:30 and 8:30 a.m.

### Laryngeal Secretion Procedure and Assessment of Inflammatory Analytes

A total of 4 secretion specimens were collected from each subject at 4 different times per treatment condition: at baseline, immediately after vocal loading, following the 4-hr treatment and 24 hours post-baseline. For baseline secretion collection, an otolaryngologist first examined the subject's oral cavity, oropharynx, and nasal cavity and placed a cotton pledget (a flat absorbent pad) soaked with lidocaine and decongestant into the subject's more patent nasal cavity. Cetacaine® was sprayed into the oropharynx. Rigid laryngeal stroboscopy was performed to obtain a baseline stroboscopic evaluation of the patient. Then 4% lidocaine was dripped onto the endolarynx through the working channel of a chip-tip flexible laryngoscope. After approximately 5 minutes, subsequent to verification of anesthesia to light touch, a one millimeter plastic cannula was passed through the working channel of the scope and guided down to the free edge and superior surface of the vocal folds while suction was applied to the catheter. That procedure allowed for the collection of a small amount of vocal fold secretions (about 100 µl), while minimizing contact of the scope with the vocal folds bilaterally. Secretions were captured in a modified sinus trap and then transferred into a 0.2 ml microfuge tube using a 1cc syringe. The tubes were labeled using codes that could not be traced to the subject or the subject's condition—except by way of a secret list retained by one investigator who was not involved with secretion data analysis–and the tubes were placed on dry ice. Tubes were then stored at −80°C until analysis.

All secretion analyses were carried out by an investigator who was blinded to subjects' conditions (time point and treatment condition). For the analyses, a known volume was aliquoted for analysis and served as the dilution factor. The appropriate volume of sterile saline was added to the tube to bring the total volume up to 2.0 ml. Standard enzyme-linked immunosorbent assays (ELISAs) were performed for IL-1β, IL-6, IL-8, TNF-α, matrix metalloproteinase (MMP)-8, and IL-10 utilizing the manufacturer's recommended protocol (R&D Systems, Minneapolis, MN). All samples were run on the same kit to avoid inter-kit variability.

Inspection of the laryngeal secretion data was carried out in order to identify the cleanest data for the development of ABM. To do so, the secretion data were sorted into three main categories for each subject and inflammatory marker: (1) data showing high baseline concentrations of pro-inflammatory markers (≥1 standard deviation from the sample mean; designated as “pre-inflamed” data); (2) data showing normal baseline concentrations of markers (<1 standard deviation from the sample mean), but paradoxically decreasing post loading (“non-responsive” data); and (3) data showing normal baseline concentrations of markers (<1 standard deviation from the sample mean) and increase after loading (“responsive” data) [35]. In addition, data from two subjects (Subject 8 and Subject 9) were considered as invalid due to thick secretions that compromised interpretation of ELISA results. Therefore, their data were not used in the development of the ABM.

As a result of this process, a base data set was identified using data from three subjects (Subject 1—voice rest, Subject 2—resonant voice, and Subject 3—spontaneous speech), whose mediator data were considered “responsive” and “not pre-inflamed”. Of note, Subject 3 was the one who participated in both the between- and within-groups design. The first three time points of the base data set were used for ABM calibration. Also, mediator data at 24 hr for (1) Subjects 1, 2 and 3 as well as (2) Subjects 4–7, whose inflammatory mediator patterns were anomalous in some fashion, was used for model validation.

### ABM Development

The ABM of inflammation and tissue damage/healing was a modification of one we developed previously to address the prototypical wound healing scenario, namely skin healing [97]. The freeware *Netlogo* (Center for Connected Learning and Computer-Based Modeling, Northwestern University, Evanston, IL) was used as the platform for model building and simulation. This first-generation ABM aimed to reproduce the basic and generally-accepted mechanisms of wound healing. Thus, detailed literature on inflammation and wound healing was reviewed to identify the essential components and rules for this ABM [36], [68]–[70], [98]. Then, experimental measures of inflammatory mediators in human laryngeal secretions [35] were used to adapt the model to the setting of vocal fold injury.

**Figure 3:**
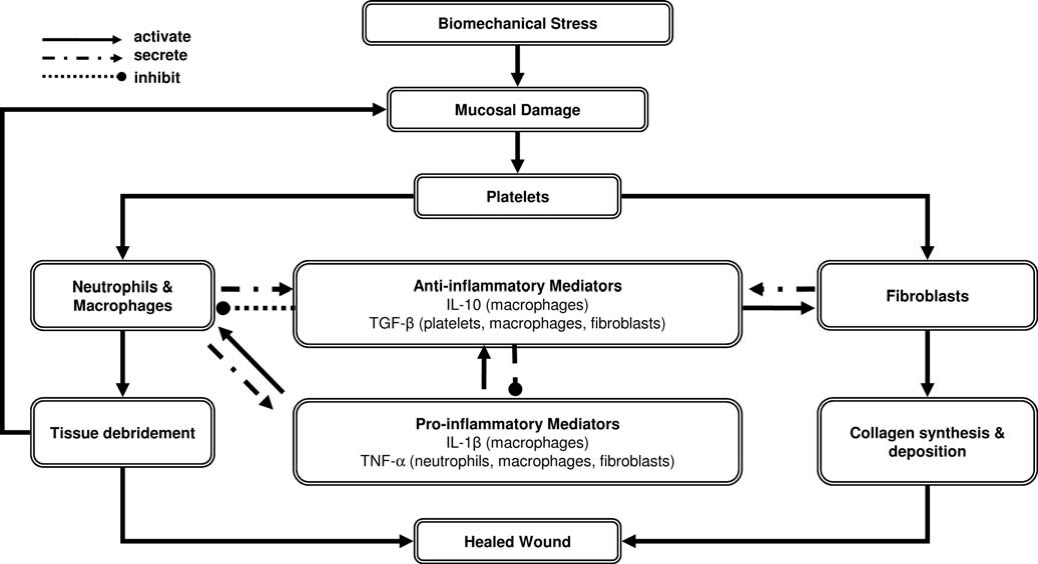
An overall flowchart of the model. The model assumes that biomechanical stress during phonation causes mucosal damage and activates platelets, neutrophils and macrophages. Platelets produce TGF-β1, which chemoattracts both neutrophils and macrophages. Activated neutrophils and macrophages secrete pro-inflammatory mediators, which in turn induce anti-inflammatory mediator release. Pro-inflammatory mediators also induce neutrophils and macrophages to produce free radicals that damage tissue. In our model, the activity of free radicals was subsumed in the actions of TNF-α. Anti-inflammatory mediators contribute to fibroblast activation. Activated fibroblasts secrete collagen that mediates tissue repair. In the model, collagen accumulation is considered as the surrogate for healing outcome following phonotrauma. Collagen is an important ECM protein involving both structural and biomechanical functions in the vocal folds (Gray & Titze, 1988; Gray et al., 2000).

Simply put, our ABM of phonotrauma represents processes thought to occur in the vocal fold mucosal tissue and to simulate the mucosal repair response to biomechanical damage during phonation. The model consists of platelets, inflammatory cells (neutrophils, macrophages, and fibroblasts), mediators that involve in inflammation and wound healing (IL-1β, TNF-α, IL-10, and TGF-β1), a representative component of the extracellular matrix (collagen), and, perhaps most important, a tissue damage function functionally analogous to alarm/danger signals [99] that produces positive feedback to induce further inflammation [67] (Figure 3; Table 1).

**Table 1:** Summary of the components involved in the ABM. The biological functions in *italics* are the extension of the new existing ABM.

Cell Sources	Substances	Biological Functions in Wound Healing used in ABM
Platelets Macrophages Fibroblasts	TGF-β1	Chemotactic to neutrophils, macrophages and fibroblasts
		Inhibit expression of TNF-α in neutrophils, macrophages and fibroblasts
		Inhibit expression of IL-1β in macrophages (minimal effect)
		Stimulate resting fibroblasts to activated fibroblasts
		Mitogenic to fibroblasts (proliferation)
		Stimulate collagen synthesis in fibroblasts
Neutrophils Macrophages Fibroblasts	TNF-α	Chemotactic to neutrophils and macrophages
		Activate neutrophils and macrophages
		Stimulate expressions of TNF-α and IL-1β in macrophages
		Stimulate expression of TGF-β in macrophages and fibroblasts
		Mitogenic to fibroblast (proliferation)
		Induce tissue damage
Macrophages	IL-1β	Chemotactic to neutrophils and macrophages
		Activate macrophages
		Stimulate expressions of TNF-α and IL-1β in macrophages
		Mitogenic to fibroblasts (proliferation)
		Inhibit collagen synthesis in fibroblasts
Macrophages	IL-10	Inhibit expression of TNF-α in neutrophils, macrophages and fibroblasts
		Inhibit expression of IL-1β in macrophages
		Stimulate expression of TGF-β in macrophages and fibroblasts
		Stimulate expression of IL-10 in macrophages
		Inhibit activated neutrophil survival
		Inhibit activation of neutrophils and macrophages
Fibroblasts	Collagen	Repair tissue damage

### Regions, patches and agents of the vocal fold ABM

A typical ABM is composed of three elements: region, patch and agent. The *region* is composed of small patches. The *patches* are immobile components that characterize the physical-spatial environment, where the *agents* operate. Agents are the active objects that move and interact within the region. In our ABM, the “world” is a square grid of 120×120 patches, with the origin in the center of the grid. Two *regions* were created to simulate (1) blood and (2) mucosal tissue itself. These two regions do not intersect. Specifically, the tissue region is a circle with a diameter of 55, centered at the origin and bounded by the blood region. The blood region is the source of the inflammatory cells that infiltrate the wounded tissue. At the same time, the region of mucosal tissue is the source of some resident cells and is also the site in which phonotraumatic injury occurs and is subsequently repaired by fibroblasts [100].

*Patch* variables were used to represent (1) tissue status (healthy, damaged, and healed); (2) platelets; (3) collagen; and (4) inflammatory mediators. Platelets are important to initiate the inflammatory process following tissue damage. The initial number of platelets is spatially distributed based on the rules in our model. Collagen is the major structural protein in the vocal folds and its content and organization are prone to be disturbed following repetitive phonotrauma [101]–[106]. In our model, the amount of collagen was required not to exceed the existing amount of damage in the same patch. Two pro-inflammatory mediators (IL-1β and TNF-α) and two anti-inflammatory mediators (IL-10 and TGF-β1) were also selected as patches because they are generally believed to play an important role in wound healing environment and because we had prior data on their expressions in the vocal folds [48]–[50]. The concentrations of the inflammatory mediators on each patch are controlled by formulae for mediator synthesis, mediator degradation and mediator diffusion.

*Agent* variables were used to represent (1) tissue damage and (2) cells. Tissue damage is induced by the initial injury and the subsequent inflammatory response of the pro-inflammatory mediators (IL-1β and TNF-α). Tissue damage also acts as a stimulus for further inflammation. Another class of agent is cells, namely, neutrophils, macrophages and fibroblasts. In our model, cells have three states: resting, activated or dead. Cells are represented as agents because they can be organized based on common behavioral rules, and because the response of a particular cell type to various mediators is readily characterized in the literature [92]. Cell behavior was governed by rules based on existing wound healing literature [36], [68]–[70], [98]. Depending on the cell type, the cellular responses included activation, migration, proliferation, cell death, secretion of inflammatory mediators, tissue debridement, and collagen generation. The complete rules of the ABM with explanations are attached in *Supporting Information (* *Table S1* *)*.

### Simulation of acute phonotrauma

For each simulation, the user can define the initial levels of IL-1β, TNF-α and IL-10, add a phonotraumatic event, and then a 4-hr treatment event (voice rest, “resonant voice” exercises or spontaneous speech). We assumed in the model that one step of simulated time represents 0.1 days or approximately 2.4 hours. The changes in temporal concentration of inflammatory cells, mediators, tissue damage and collagen were plotted and refolded into the model at each time step.

Initially, some resting neutrophils are in the blood region, whereas some macrophages and fibroblasts are present with a random distribution in both blood and tissue regions. Simulated phonatory stresses traumatized the mucosal tissue in the middle of region, triggering platelet degranulation. Shortly afterwards, a chemoattractant gradient is created that stimulates the infiltration and activation of neutrophils and macrophages. Later on, fibroblasts are activated by tissue damage and TGF-β1. Fibroblasts secrete collagen to repair both the initial and the inflammation-induced damage. Last, additional mechanical stresses are applied to the traumatized tissue based on the treatment selected (voice rest: no additional mechanical stress; resonant voice: low mechanical stress; spontaneous speech: high mechanical stress).

### Model Calibration and Validation

Standard procedures to evaluate the fit of ABM to empirical data have not been established in the literature. In the present study, pattern-oriented analysis [107] was used to estimate the conformity of simulation-generated data curves with the inflammatory and wound healing patterns reported in the literature as well as the empirical data sets around acute phonotrauma (Table 2).

**Table 2:** Patterns used for ABM at the comparison condition, i.e., the mid-point of the magnitude of initial mechanical stress input.

Patterns of Inflammation and Healing	Resource
Neutrophils arrive in wound site in the first few hours	[68]–[70], [98]
Neutrophil number is at maximum by 24 hours	[68]–[70], [98]
Neutrophil number decreases rapidly on Day 3	[68]–[70], [98]
Macrophage number is at maximum by 24–48 hours	[68]–[70], [98]
Fibroblast number is at maximum by Day 5–7	[68]–[70], [98]
Fibroblast number decreases gradually on Day 7	[68]–[70], [98]
Collagen curve is sigmoid-shaped	[70], [98]

Using this approach, the user-defined initial magnitude of mucosal injury (range 0–40 in arbitrary units of damage) was set at a value of 20 as a “comparison condition”, because that setting resulted in realistic predictions of mucosal damage and healing when compared with the general consensus around laryngeal wound healing documented in the literature [68]–[70], [98]. When the qualitative behavior of the simulation appeared satisfactory, we proceeded to calibrate the model by adjusting parameter values not found in the literature to fit the quantity and time-course of measured vocal fold mediators. We calibrated the ABM using data from the base cohort (Subjects 1, 2 and 3) using their baseline mediator levels in laryngeal fluid, immediately after phonotrauma induction, and following a 4-hr treatment (voice rest, “resonant voice” exercises, or spontaneous speech) (Figures 1–2, dark circles) [35]. Subsequently, we validated the ABM quantitatively by comparing the predicted mediator levels with the empirical mediator levels at 24 hr (1) for Subjects 1, 2 and 3, as well as (2) Subjects 4–7 whose data had never been used for the model.

Due to the inherent stochasticity of the ABM framework, we performed ten runs of the calibrated ABM for each subject up to five simulated days under the condition of (1) each subject's actual treatment group and (2) hypothetical randomization to either of the other two treatment groups for all subjects, i.e., hypothetical treatments. The means and standard deviations of model variables (concentrations of inflammatory cells, mediators, and tissue damage) were computed at each time point for subsequent analysis. Hypothetical treatments were included for simulation to demonstrate the application of ABM for running individual-specific *in silico* clinical trials.

### Statistical Analysis

Z-tests using the *p-value* approach to testing statistical significance were carried out to compare the human empirical data and the data predicted by the model for IL-1β, TNF-α and IL-10 at the 24-hr time point for (1) the base cohort (Subjects 1–3) and (2) Subjects 4–7. Also, linear mixed models with fixed and random effects were used to compare the predicted levels of IL-1β, TNF-α, and IL-10. Fixed factors included: (1) *patient*, (2) *treatment condition* (spontaneous speech, voice rest and “resonant voice” exercises) and (3) *time point* (immediately after phonotrauma induction, following a 4-hr treatment and 24-hr post baseline). The random factor is *the number of simulation runs* and is nested within the *patient* factor.

## Supporting Information

Table S1 ABM RULES.
(0.19 MB RTF)
