# Supplementary material for: A Patient-Specific in silico Model of Inflammation and Healing Tested in Acute Vocal Fold Injury
Source: PLoS One. 2008 Jul 30;3(7):e2789. doi: 10.1371/journal.pone.0002789 (PMC2481293; doi:10.1371/journal.pone.0002789)
Supplement: Table S1 — ABM RULES. (0.19 MB RTF) [file pone.0002789.s001.txt]

Table S1: ABM RULES

Parameter Descriptions	Rules	
Extent of mucosal damage created by custom-defined magnitude	Initial damage = Magnitude * Magnitude + a normally distributed random number with mean = 0 and standard deviation = 1000	
		
Extent of mucosal damage induced by TNF-	If TNF->0.1, damage + 1	
		
Effect of the 4-hr resonant voice exercise on mucosa	If the time step is between 1 and 3, create a damage = 10 + a normally distributed random number with mean = 0 and standard deviation = 10	
		
Effect of the 4-hr spontaneous speech on mucosa	If the time step is between 1 and 3, create a damage = 30 + a normally distributed random number with mean = 0 and standard deviation = 10	
		
TGF-1 secreted by platelets	TGF-1 = TGF-1 + number of platelet/10	
		
Platelet diffusion coefficient	Platelet = 1 unit/step	
		
Inflammatory mediator diffusion speed 	IL-1 = 1 unit/step
TNF- = 1 unit/step
TGF-1 = 1 unit/step
IL-10 = 1 unit/step	
		
Collagen diffusion coefficient	0.5 unit/step	
		
Inflammatory mediator degradation speed	IL-1 = 0.2 unit/step
TNF- = 0.2 unit/step
TGF-1 = 0.2 unit/step
IL-10 = 0.25 unit/step	
		
Collagen degradation coefficient	0.99 unit/step	
		
Collagen repairs tissue damage	If collagen>1, the damaged tissue that underneath is repaired by the collagen.	
		
Initial number of neutrophils	60	
		
Number of neutrophils recruited relating to tissue damage	2 * (damage / magnitude2 ) + 1	
		
		
Platelets chemoattract neutrophils 	If plateletright>plateletahead and plateletright>plateletleft, 
the neutrophil turns 90 right. 
If plateletleft>plateletright, the neutrophil turns 90 left. 	
		
TNF- chemoattracts neutrophils	If TNF-right> TNF-ahead and TNF-right> TNF-left, the neutrophil turns 90 right. 
If TNF-left> TNF-right, the neutrophil turns 90 left. 	
TGF-1 chemoattracts neutrophils	If TGF-1right> TGF-1ahead and TGF-1right> TGF-1left, the neutrophil turns 90 right. 
If TGF-1left> TGF-1right, the neutrophil turns 90 left. 	
		
IL-1 chemoattracts neutrophils	If IL-1right> IL-1ahead and IL-1right> IL-1left, the neutrophil turns 90 right. 
If IL-1left> IL-1right, the neutrophil turns 90 left. 	
		
Area of mucosal damage that activates neutrophils	If the neutrophil migrates to the area that has the damage greater than (magnitude + total-damage * 2), the neutrophil is activated.	
		
TNF- (and IL-10)  stimulates (and inhibits) activation of neutrophils	If TNF- - (IL-10 * 5000) > 0.1, the neutrophil is activated.	
		
TNF- secreted by activated neutrophil	TNF- =  TNF-  + (0.1 * ( 1 / (TGF-1 * 0.5 +  IL-10 * 5) )	
		
Neutrophil lifespan	2 to 4 days	
		
IL-10 inhibits activated neutrophil survival	If total-IL-10 > 1, the neutrophil age – 0.5	
		
Initial number of residential macrophages 	120	
		
Magnitude of damage to recruit resting macrophage 	If damage > magnitude * 1.2, resting macrophage will be recruited. 	
		
Number of macrophages recruited  relating to damage	4 * damage / magnitude2  +  1	
		
Platelets chemoattract macrophages	If plateletright>plateletahead and plateletright>plateletleft, 
the macrophage turns 90 right. 
If plateletleft>plateletright, the macrophage turns 90 left. 	
TNF- chemoattracts macrophages	If TNF-right> TNF-ahead and TNF-right> TNF-left, 
the macrophage turns 90 right. 
If TNF-left> TNF-right, the macrophage turns 90 left. 	
		
TGF-1 chemoattracts macrophages	If TGF-1right> TGF-1ahead and TGF-1right> TGF-1left, the macrophage turns 90 right. 
If TGF-1left> TGF-1right, the macrophage turns 90 left. 	
		
IL-1 chemoattracts macrophages	If IL-1right> IL-1ahead and IL-1right> IL-1left, the macrophage turns 90 right. 
If IL-1left> IL-1right, the macrophage turns 90 left. 	
		
Area of mucosal damage that activates macrophages	If the macrophage migrates to the area that has the damage greater than (magnitude + total-damage) * 0.01, the macrophage is activated.	
		
IL-1,TNF- (and IL-10) stimulates (and inhibits) activation of macrophages	If IL-1 + TNF- - IL-10 > 400, the macrophage is activated.	
		
Inflammatory mediators secreted by activated macrophages under voice rest condition	TNF- = TNF- + 0.1 * (1 / (1 + TGF-1 + total-IL-10)) * (1 + total-TNF-+ total-IL-1 +40)
IL-1 = IL-1 + 1 * (1 / (1 + TGF-1 + total-IL-10)) * (1 + total-TNF- * 30 + total-IL-1 + 40)
IL-10 = IL-10 + 2 + (total-IL-10 * 0.001)
TGF-1 = TGF-1 + (1+ total-IL-10 + total-TNF-)	
		
Inflammatory mediators secreted by activated macrophages under resonant voice condition	TNF- = TNF- + 0.1 * (1 / (1 + TGF-1 + total-IL-10)) * (1 + total-TNF-+ total-IL-1 * 0.5 +40 )
IL-1 = IL-1 + 1 * (1 / (1+ TGF-1 + total-IL-10)) * (1 + total-TNF- * 10 + total-IL-1 + 40)
IL-10 = IL-10 + 1 + (total-IL-10 * 0.01)
TGF- = TGF- + (1+ total-IL-10 + total-TNF-)	
		
Inflammatory mediators secreted by activated macrophages under spontaneous speech condition	TNF- = TNF- + 0.1 * (1 / (1 + TGF-1 + total-IL-10)) * (1 + total-TNF-+ total-IL-1 * 0.5 +40)
IL-1 = IL-1 + 1 * (1 / (1+ TGF-1 * 0.8 + total-IL-10)) * (1 + total-TNF- * 50 + total-IL-1 + 40)
IL-10 = IL-10 + 1.5 + (total-IL-10 * 0.001)
TGF-1 = TGF-1 + (1+ total-IL-10 + total-TNF-)	
		
Time period that macrophages may kill neutrophils	During 3.8-4.8 day post-jury	
Probability of macrophages killing neutrophils	90% chance after 4-5 post-damage days	
		
Macrophage lifespan	5-9 days	
		
Initial number of residential fibroblasts	250	
		
Magnitude of damage to recruit tissue fibroblasts 	If damage > magnitude * 1.2, tissue fibroblasts will be recruited.	
		
Number of tissue fibroblasts being recruited related to damage	4 * damage / magnitude2  +  1	
		
TGF-1 chemoattracts tissue fibroblasts	If TGF-1right> TGF-1ahead and TGF-1right> TGF-1left, the tissue fibroblast turns 90 right. 
If TGF-1left> TGF-1right, the tissue fibroblast turns 90 left. 	
		
Tissue fibroblasts differentiate to activated fibroblasts	If the tissue fibroblast migrates to the area that has the damage greater than TGF-1 > 0.1, the tissue fibroblast differentiates to activated fibroblast.	
		
Proliferation of activated fibroblasts	If the activated fibroblast ages 3-13 days, the fibroblast proliferates.	
		
Fibroblast proliferation are stimulated by IL-1, TNF- and TGF-1	1 + 0.001 * (total-TGF-1 * 0.5 + total-TNF * 0.5 + total-IL-1 * 0.5)	
		
Inflammatory mediators secreted by activated fibroblasts	TNF-á = TNF-á + 0.2 * (1 / (1 + total-TGF-1 + total-IL-10))
TGF-1 = TGF-1 + (1 + total-TNF-á * 0.5 + total-IL-10 * 0.5 )	
		
Collagen secreted by activated fibroblasts	Collagen = Collagen + 2 * (1 + total-TGF-1 * 2) / (1 + total-IL-1 * 0.5)	
		
Fibroblast lifespan	3-5 days	
